# Differential responses of cryptic bat species to the urban landscape

**DOI:** 10.1002/ece3.1996

**Published:** 2016-02-26

**Authors:** Paul R. Lintott, Kate Barlow, Nils Bunnefeld, Philip Briggs, Clara Gajas Roig, Kirsty J. Park

**Affiliations:** ^1^ College of Life and Environmental SciencesUniversity of ExeterDevonEX4 4PSU.K.; ^2^ Bat Conservation TrustQuadrant House250 Kennington LaneLondonSE11 5RDU.K.; ^3^ Biological and Environmental SciencesSchool of Natural SciencesUniversity of StirlingStirlingFK9 4LAU.K.

**Keywords:** Bats, conservation, cryptic species, land use, population trends, urban ecology

## Abstract

Urbanization is a key global driver in the modification of land use and has been linked to population declines even in widespread and relatively common species. Cities comprise a complex assortment of habitat types yet we know relatively little about the effects of their composition and spatial configuration on species distribution. Although many bat species exploit human resources, the majority of species are negatively impacted by urbanization. Here, we use data from the National Bat Monitoring Programme, a long‐running citizen science scheme, to assess how two cryptic European bat species respond to the urban landscape. A total of 124 × 1 km^2^ sites throughout Britain were surveyed. The landscape surrounding each site was mapped and classified into discrete biotope types (e.g., woodland). Generalized linear models were used to assess differences in the response to the urban environment between the two species, and which landscape factors were associated with the distributions of *P. pipistrellus* and *P. pygmaeus*. The relative prevalence of *P. pygmaeus* compared to *P. pipistrellus* was greater in urban landscapes with a higher density of rivers and lakes, whereas *P. pipistrellus* was frequently detected in landscapes comprising a high proportion of green space (e.g., parklands). Although *P. pipistrellus* is thought to be well adapted to the urban landscape, we found a strong negative response to urbanization at a relatively local scale (1 km), whilst *P. pygmaeus* was detected more regularly in wooded urban landscapes containing freshwater. These results show differential habitat use at a landscape scale of two morphologically similar species, indicating that cryptic species may respond differently to anthropogenic disturbance. Even species considered relatively common and well adapted to the urban landscape may respond negatively to the built environment highlighting the future challenges involved in maintaining biodiversity within an increasingly urbanized world.

## Introduction

Over the past two centuries, rapid urban expansion has become a dominant driving force within global environmental change (Wu et al. 2013). Urban areas represent unique combinations of disturbances, stresses, structures, and functions (Pickett et al. 1997), and relatively little is known of how to maintain or manage wildlife within urban ecosystems (Shwartz et al. 2014). The degree to which a landscape can facilitate or restrain movement of organisms amongst resource patches (“connectivity”) is a critical factor on dispersal rates, home range movements, colonization rates, and extinction risk and hence influences species distributions (Tischendorf and Fahrig 2000). A landscape‐scale approach is therefore needed to understand how the composition, configuration, and spatial heterogeneity of the urban landscape impacts upon species persistence within the built environment.

Urbanization imposes stresses that relatively few species are able to adapt to (Ditchkoff et al. 2006). Examining how species respond to urbanization enables us to identify those species which may require most conservation effort to cope with anthropogenic disturbances. Morphological or behavioral factors can influence how adept certain species are at adapting to urbanization. These traits have therefore been used to classify species as “urban avoiders,” “urban utilizers,” or “urban dwellers” (Fischer et al. 2015), although in reality, there is likely to be a continuous spectrum of adaptability. Given the scarcity of information on species‐specific responses to urbanization, the likely response of an individual species to the urban landscape is often predicted from its morphological traits (e.g., Jung and Kalko 2011; Threlfall et al. 2012). Such congruence in response to urbanization would suggest that species‐specific conservation strategies would also benefit morphologically similar species, although this has rarely been tested (but see Lintott et al. 2015).

Although many species of Chiroptera (bats) have formed strong associations with human settlements (e.g., roosting in buildings; Jenkins et al. 1998), the general pattern is of lower bat activity and species richness with increasing levels of urbanization (e.g., Gaisler et al. 1998; Lane et al. 2006). The loss and fragmentation of natural and semi‐natural habitats within the urban landscape has reduced the availability of foraging grounds for bats (Russo and Ancillotto 2014). Additionally, movement within the built environment will frequently involve flying over busy roads which can be a major source of bat mortality and anthropogenic disturbances (e.g., noise and light pollution) which can exclude bats from foraging resources (Stone et al. 2009; Lesiński et al. 2011; Berthinussen & Altringham 2012).

Most conservation effort is focused on already vulnerable species; however, there is increasing evidence that some widespread species are also declining rapidly and that changes in land use are the primary driver for this (Shreeve and Dennis 2011). Here, we study two, often sympatric, cryptic species of pipistrelle *Pipistrellus pygmaeus* and *P. pipistrellus* which, although relatively widespread across Europe, are thought to have experienced historic population declines, (Stebbings 1988), although there is evidence of a modest recovery more recently (Barlow et al. 2015). Only formally recognized as different species as recently as 1999 (Jones and Barratt 1999), these two species are morphologically similar and adopt comparable foraging strategies (Barlow and Jones 1997; Nicholls and Racey 2006a). In relation to their foraging activity in urban landscapes, Hale et al. (2012) found that peak *P. pipistrellus* activity occurred at ponds surrounded by moderate levels of urban infrastructure, whereas Lintott et al. (2015) found differences between the two species, with *P. pygmaeus* more likely to be found in woodlands with low clutter and understory cover which were surrounded by low levels of built environment. However, these studies were conducted at local or regional scales and focused on specific habitats in urban areas (e.g., ponds in the West Midlands, U.K. – Hale et al. 2012; woodlands in central Scotland – Lintott et al. 2015). Here, we take a landscape‐scale approach using data from the National Bat Monitoring Programme (NBMP), a long‐running citizen science scheme (see http://www.bats.org.uk/pages/nbmp.html; Barlow et al. 2015) to determine how these two species respond to urban landscapes in towns and cities throughout Great Britain. In particular, we address the following questions:

1. Do two cryptic, morphologically similar species respond to the composition and spatial configuration of urban landscape in a similar manner?
2. How do the composition, configuration, and heterogeneity of the urban landscape influence the distribution of two widespread and relatively common species of bat on a large (i.e., country‐wide) scale?

We then discuss the conservation implications of these findings and highlight the importance of meeting the conservation needs of common species that are frequently overlooked given their abundance and widespread distribution.

## Methods

### Site selection

**Figure 1:**
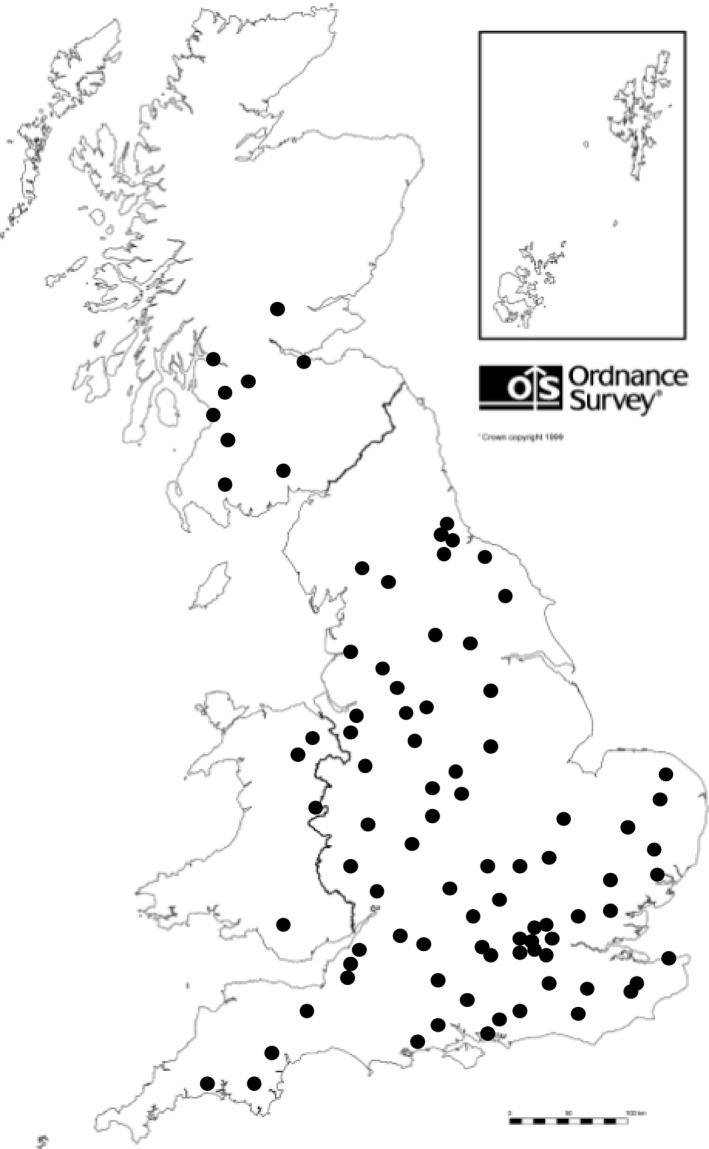
The location of the 124 urban transects undertaken as part of the Bat Conservation Trust's National Bat Monitoring Programme. Reproduced from Ordnance Survey map data by permission of Ordnance Survey © Crown copyright 2013.

This study focuses on the response of species to the built environment; therefore, only sites classified as urban were included, although the % cover of gray space (e.g., buildings and roads) within a radius of 1 km varied widely from 1% to 67%). Urban areas were designated as those where urban cover was the dominant land use within a 1‐km grid square as categorized by Boughey et al. (2011). Sites were selected which had been surveyed for at least 2 years between 2007 and 2012 (surveys conducted prior to 2007 were discounted given the rapid land use change that occurs in cities) and were a minimum of 5 km apart to minimize the possibility of sampling the same population of bats. This resulted in a total of 124 urban sites surrounded by a wide diversity of landscapes (Fig. 1, Appendix S1).

Field surveys were conducted annually by trained volunteer surveyors in suitable weather conditions (avoiding heavy rain, high winds, and temperatures at sunset below 7°C; Barlow et al. 2015). Surveyors conducted two surveys (a minimum of 5 days apart) in July following an approximately triangular transect (3 km in length) within a randomly allocated 1 km grid square. Surveyors undertook 2‐min point counts at 12 evenly spaced locations where a heterodyne bat detector was tuned to 50 kHz and the number of bat passes (a continuous sequence of echolocation calls) of *P. pipistrellus* and *P. pygmaeus* was counted. Although it has been shown the accurate species identification is possible for a wide range of European bat species using heterodyne bat detectors (Ahlen and Baagøe 1999), some error in bat identification is still likely (Barlow et al. 2015). Therefore, volunteers were provided with the option of including an “Unsure pipistrelle” count for those bat calls which they heard but were unable to identify to species level with any certainty (for full details of the survey methods, see Barlow et al. 2015).

### Landscape analysis

We plotted transects using ArcGIS 10 (ESRI Inc 2013) to determine the center point of the 12‐point counts within each site. Buffers of 1, 2, and 3 km were created around the central point reflecting the upper limit of home range size for *P. pygmaeus* and *P. pipistrellus* (Nicholls and Racey 2006b). We used data from the OS MasterMap Topography Layer (EDINA Digimap Ordnance Survey Service 2013) to reclassify the landscape within each buffer into a set of discrete biotope types. These were (1) gray space (buildings, structures, roads, and paths); (2) green space (gardens, parkland, managed grassland, rough grassland, and farmland); (3) inland fresh water; and (4) woodland (coniferous, deciduous, and mixed woodland). A measure of connectivity within the urban landscape, the woodland Euclidean nearest neighbor distance (ENN, the mean value of ENN distances amongst all woodland patches within the landscape) and the Shannon diversity index (SHDI, a measure of landscape heterogeneity) was calculated as previous studies have found these variables to influence bat foraging activity (e.g., Lintott et al. 2014). We calculated the proportion of land covered by each biotope, woodland ENN, and SHDI for each buffer scale using Fragstats v4.0 (McGarigal et al. 2002).

### Data analysis

Statistical analyses were undertaken using R version 2.14 (R Core Team 2012) using the lme4 (Bates et al. 2013) and effects package (Fox 2003).

#### Differences in the response to the urban environment by two cryptic bat species

We performed a generalized linear model (GLM) with binomial error distribution and a logit link to quantify the influence of the urban matrix in the presence of *P. pipistrellus* and *P. pygmaeus*. To assess the relative effect of the surrounding landscape on *P. pygmaeus* in comparison with *P. pipistrellus*, the model was run with the response variable expressed as the proportion of the number of point counts per transect where *P. pygmaeus* was recorded versus the number of point counts per transect where *P. pipistrellus* was recorded. Calls that volunteers categorized as “unsure pipistrelle” were dropped from further analysis (12% of all pipstrelle calls recorded). As high collinearity is found amongst landscape metrics (i.e., between the proportions of various biotope types or the same biotope type at a range of spatial scales), we undertook preliminary GLMs to determine which metrics at which spatial scale should be included in the model; metrics with the highest *R* ^2^ value and lowest AIC value were selected for inclusion in the final model (Appendix S2). When several landscape parameters seemed equally important (i.e., <5% difference from the highest R^2^ value), they were all selected providing they were not strongly correlated (Pearson correlation coefficient <0.4 and *P* > 0.05 used as thresholds). We also included the easting and northing Cartesian coordinates of each transect into the model as the population densities of the two species vary across the U.K. (Altringham 2014). Spatial auto‐correlation was assessed using a spline correlogram of the model residuals and Moran's *I* test and was not significant (using a threshold of *α* = 0.05, Observed: 0.03, Expected −0.03, *P* = 0.06).

The results of the full model are presented including the standardized parameters and confidence intervals for each of the explanatory variables. We made inferences on the effect of each parameter by (1) contrasting its standardized estimate to other predictor variables to assess relative importance, (2) determining the upper and lower 95% quantiles of each parameter distribution which was obtained from *N* = 2000 simulated draws from the estimate distribution (following Gelman and Hill 2007), and (3) performing likelihood ratio tests to compare models by excluding each parameter in turn (Faraway 2005). Simulated draws (*n* = 2000) were undertaken to construct prediction plots from the estimated distribution of an explanatory variable, whilst all other model parameters were maintained at their median observed values (Fig. 1).

#### The impact of urbanization on common bat species

In addition to directly testing whether *P. pipistrellus* and *P. pygmaeus* respond differently to the urban landscape (Differences in the response to the urban environment by two cryptic bat species), we were interested in assessing what landscape factors were important in influencing the distributions of *P. pipistrellus* and *P. pygmaeus*. We therefore undertook two generalized linear models with negative binomial distributions, one for *P. pipistrellus* and the other for *P. pygmaeus* to determine how the urban landscape influences each of their distributions. The percentage of point counts per transect where either *P. pipistrellus* or *P. pygmaeus* were recorded was used as a measure of the relative prevalence of that species at that site. We used the same approach to determine influential explanatory variables as described in Differences in the response to the urban environment by two cryptic bat species.

## Results

The presence of *P. pipistrellus* was recorded in 117 of the 124 sites (94%) and within 27% of all point counts, whilst *P. pygmaeus* was recorded in 79 of the sites (63%) and within 12% of the point counts.

### Differences in the response to the urban environment by two cryptic bat species

In the results described below (Differences in the response to the urban environment by two cryptic bat species), it should be noted that significant variables derived from the bat GLMs indicate a differential response between the species to landscape characteristics; variables which are similarly influential for both species will not therefore be statistically significant in these models.

**Figure 2:**
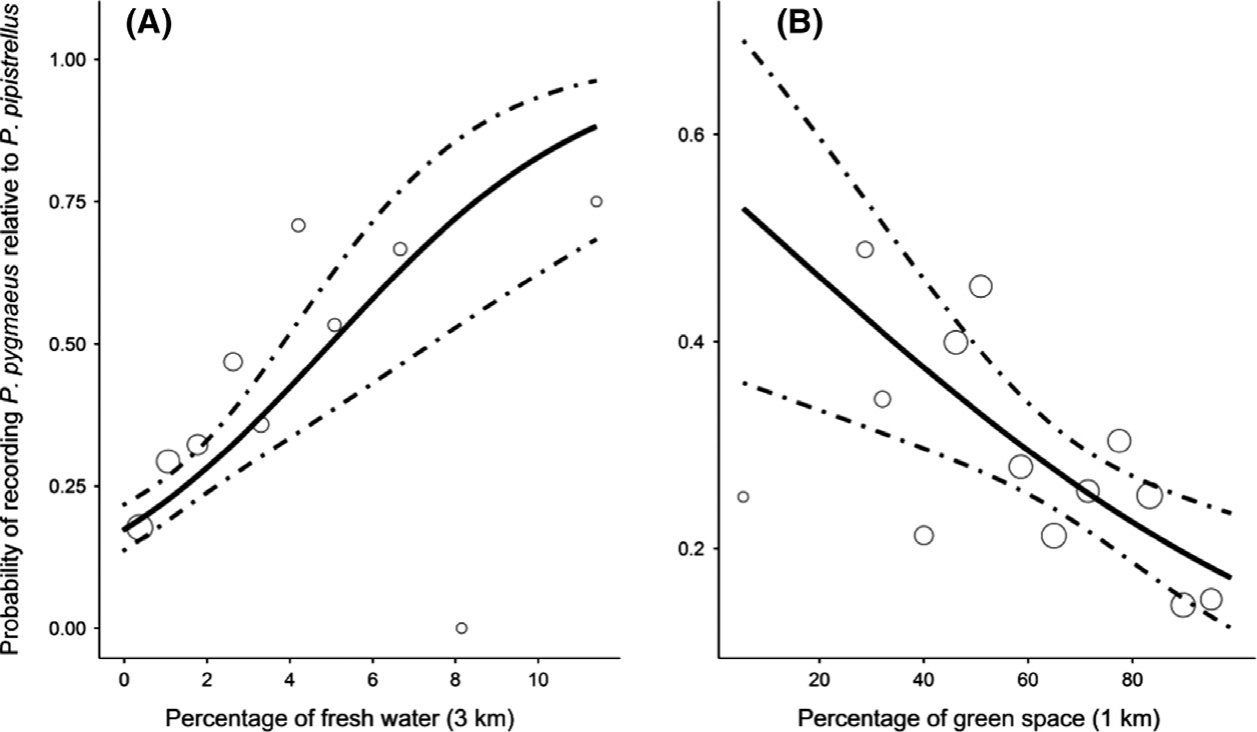
Estimated probability of recording *P. pygmaeus* relative to *P. pipistrellus* within urban landscapes. Dashed lines indicate 95% confidence intervals. Raw data on the probability of recording *P. pygmaeus* relative to *P. pipistrellus* are superimposed as gray circles with diameter proportional to the total number of sites where either species was recorded.

Based on the estimated coefficients in Table 1, in locations with very few rivers or lakes in the surrounding 3 km, there was a 0.17 (95% CI: 0.14–0.22) probability of recording *P. pygmaeus* relative to *P. pipistrellus*; conversely *P. pygmaeus* was more likely to be recorded (0.72; 0.53–0.86) in locations containing higher proportions (8%) of freshwater (Fig. 2A). *P. pygmaeus* and *P. pipistrellus* were equally likely to be recorded in urban areas with low levels of green space in the surrounding 1 km (20%), whilst the probability of recording *P. pygmaeus* relative to *P. pipistrellus* reduced to 0.23 (0.19–0.27) in urban areas comprising a high proportion of green space (80%; Fig. 2B). *P. pygmaeus* were also more likely to be recorded in landscapes with higher woodland connectivity in the surrounding 3 km (Table 1); however, the relationship was strongly influenced by one outlier which, when excluded, substantially reduced the effect of this variable. The inclusion, or exclusion, of the outlier had little influence on overall model fit or any other variable.

**Table 1:** Parameter estimates and likelihood ratio tests of GLM for the probability of detecting *P. pygmaeus* relative to *P. pipistrellus* in urban landscapes. The model was run to calculate the probability of recording *P. pygmaeus* presence relative to *P. pipistrellus*; hence, positive estimates indicate an increased probability of detecting *P. pygmaeus,* and negative estimates indicate an increased probability of detecting *P. pipistrellus* with a given explanatory variable. Significant explanatory variables are highlighted in bold

Explanatory variable	Estimate (±SE)	Log likelihood	*χ* ^2^	*P*
Intercept	−1.01 ± 0.10			
**Proportion of freshwater (3 km)**	**0.52 ± 0.10**	−**163.47**	**23.05**	**<0.001**
**Proportion of green space (1 km)**	−**0.34 ± 0.10**	−**156.91**	**9.93**	**0.002**
**Woodland connectivity (3 km)**	**0.31 ± 0.18**	−**154.14**	**4.4**	**0.036**
Easting	−0.19 ± 0.11	−153.16	2.43	0.12
Northing	0.17 ± 0.10	−152.94	1.2	0.16

### The impact of urbanization on common bat species

**Figure 3:**
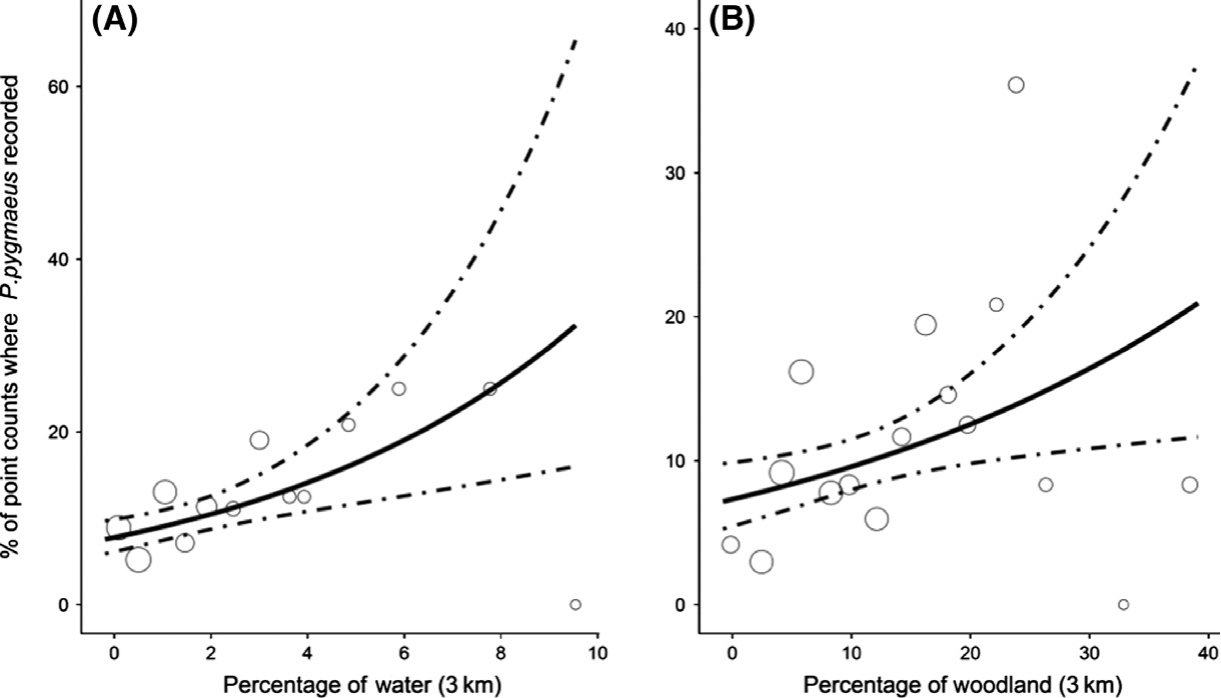
The estimated probability of recording *P. pygmaeus* in relation to the percentage of water (A) and woodland (B) in the surrounding 3 km. The size of the circles is proportional to the number of locations where *P. pygmaeus* was recorded. Dashed lines represent 95% confidence intervals around the predictions.

The number of point counts per survey where *P. pygmaeus* was recorded was positively related to the percentage of freshwater and woodland in the surrounding 3 km. In urban areas containing a relatively high percentage of freshwater (10%), the likelihood of recording *P. pygmaeus* was 0.32 (0.15–0.67) which decreased to 0.06 (0.06–0.08) in areas containing no freshwater (Fig. 3A). In locations containing no woodland, there was a low likelihood of detecting *P. pygmaeus* (0.07; 0.50–0.10), whereas the probability increased to 0.18 (0.11–0.30) in relatively wooded areas (30%; Table 2; Fig. 3B).

**Table 2:** Parameter estimates and likelihood ratio tests of GLMs for the probability of detecting either *P. pygmaeus* or *P. pipistrellus* in urban landscapes for the most important landscape parameter at the most important spatial scale. Significant explanatory variables are highlighted in bold

Species	Explanatory variable	Estimate (±SE)	Log likelihood	*χ* ^2^	*P*
*P. pygmaeus*	Intercept	0.19 ± 0.10			
***P. pygmaeus***	**Proportion of freshwater (3 km)**	**0.27 ± 0.06**	−**370.10**	**14.59**	**<0.001**
***P. pygmaeus***	**Proportion of woodland (3 km)**	**0.21 ± 0.08**	−**361.46**	**5.95**	**0.01**
*P. pygmaeus*	Easting	−0.11 ± 0.10	−357.43	1.10	0.30
*P. pygmaeus*	Northing	0.13 ± 0.10	−372.19	1.92	0.17
*P. pipistrellus*	Intercept	1.17 ± 0.05			
*P. pipistrellus*	Landscape heterogeneity (3 km)	0.05 ± 0.07	−495.93	3.39	0.18
***P. pipistrellus***	**Proportion of gray space (1 km)**	−**0.28 ± 0.08**	−**505.81**	**13.26**	**<0.001**
***P. pipistrellus***	**Proportion of freshwater (3 km)**	−**0.20 ± 0.07**	−**502.96**	**10.42**	**0.001**
*P. pipistrellus*	Easting	0.01 ± 0.06	−492.55	0.01	0.98
*P. pipistrellus*	Northing	0.01 ± 0.06	−492.55	0.01	0.95

**Figure 4:**
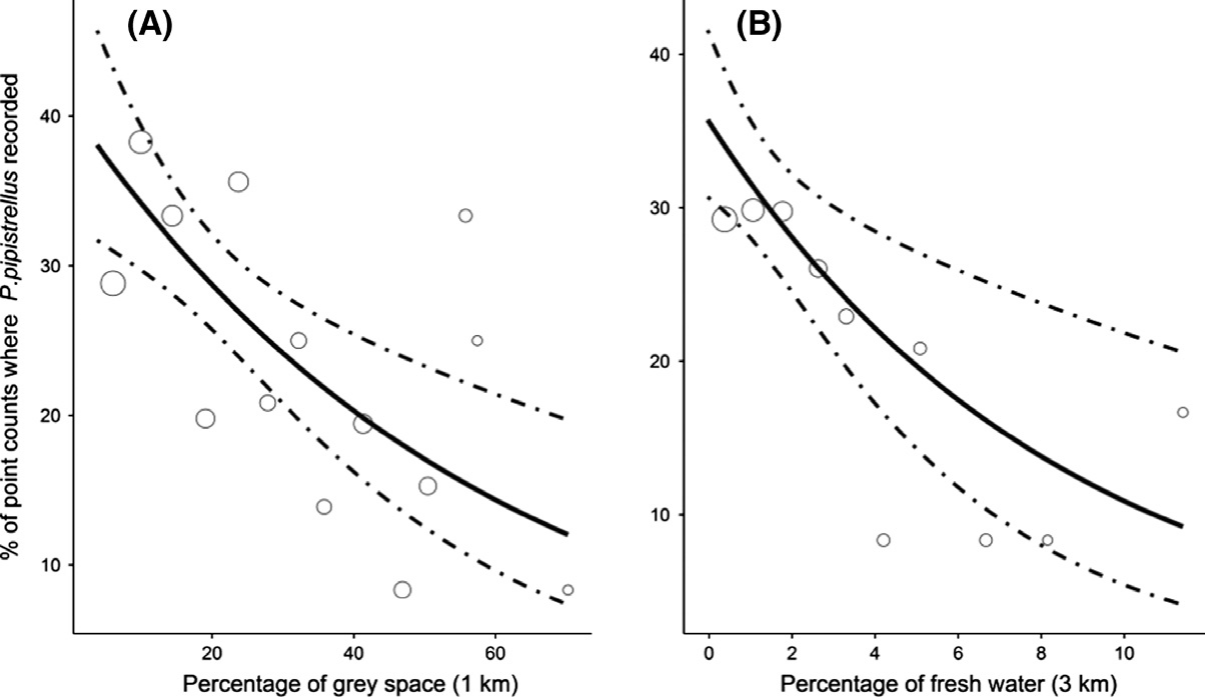
The estimated probability of recording *P. pipistrellus* in relation to the percentage of gray space in the surrounding 1 km (A) and the percentage of freshwater in the surrounding 3 km (B). The size of the circles is in proportion to the number of locations where *P. pipistrellus* was recorded. Dashed lines represent 95% confidence intervals around the predictions.

In landscapes containing low levels of gray space (5%), there was a 0.35 (0.30–0.40) probability of recording *P. pipistrellus;* however, this was reduced to 0.14 (0.90–0.22) in highly urbanized landscapes (60%; Fig. 4A). In urban areas containing no freshwater in the surrounding 3 km, the likelihood of recording *P. pipistrellus* was 0.35 (0.30–0.42) which decreased to 0.11 (0.05–0.23) in areas containing a relatively high proportion of freshwater (10%; Fig. 4B).

## Discussion

The sensitivity of bats to habitat fragmentation and changes in land use is one of many factors which have led to the recognition of bats as useful bioindicators (Russo and Jones 2015). European bat populations are showing signs of recovery, as threats such as water pollution and deliberate persecution have become less influential because of EU wide nature conservation protection measures (Van der Meij et al. 2014). However, as urbanization across Europe is projected to increase up until at least 2050 (United Nations 2014), we show that even species perceived to be relatively common and tolerant of the urban landscape respond negatively to the built environment indicating the challenges involved in maintaining biodiversity within an increasingly urbanized world.

### Differences in habitat use between *P. pipistrellus* and *P. pygmaeus*

Understanding the factors influencing the distribution of species within the built environment is critical in identifying how adaptable species are to urbanization. Species with similar morphological traits are frequently inferred to respond similarly in their response to anthropogenic environments (Safi and Kerth 2004). Our results, however, show differences in responses to the urban landscape between species which are morphologically very similar. We found that the relative prevalence of *P. pygmaeus* compared to *P. pipistrellus* was greater in landscapes with higher amounts of freshwater within the urban matrix. This supports previous studies strongly associating *P. pygmaeus* with water and riparian woodland (Oakley and Jones 1998; Nicholls and Racey 2006b), whilst *P. pipistrellus* is regarded as a generalist which can tolerate moderate levels of urbanization (Hale et al. 2012). Urban waterways facilitate the movement of species through the urban matrix (e.g., Rouqette et al. 2013); therefore, as *P. pygmaeus* is perceived to be less tolerant of the built environment (e.g., Hale et al. 2012), it is likely that waterways are one of the few habitat types that this species is using as either a foraging resource or for commuting through the urban matrix.

We found that *P. pipistrellus* was less likely to be found in locations with relatively high amounts of freshwater in the surrounding landscape. Previous studies have suggested that *P. pipistrellus* appear to actively avoid *P. pygmaeus* foraging sites resulting in differential habitat use (Nicholls and Racey 2006b; Lintott et al. 2015). Coexisting species frequently use different foraging locations to avoid excessive competition (Li et al. 2014). The low prevalence of *P. pipistrellus* in locations containing a high proportion of water may reflect that this species, as a habitat generalist, is able to use a wide variety of habitat types compared to *P. pygmaeus*. Similarly, *P. pipistrellus* was frequently recorded in urban landscapes containing a high proportion of green space (e.g., gardens, parkland, and rough grassland), supporting previous findings that *P. pipistrellus* appears to be a habitat generalist (e.g., Davidson‐Watts et al. 2006; Nicholls and Racey 2006b).

### The impact of urbanization on common bat species

Although *P. pipistrellus* is thought to be relatively well adapted to the urban landscape (Hale et al. 2012), our results indicate that it shows a strong negative response to relatively local (1 km) areas of gray space. As the rate of housing projects and developments continue to accelerate within cities, the remaining green space is becoming increasingly threatened. Our results indicate that even one of the most adaptable of European bat species may not be able to tolerate highly urbanized locations. The strong association of *P. pygmaeus* to woodland and freshwater is unsurprising as *P. pygmaeus* are well adapted to foraging along waterways, woodland edges, and within open woodland (Kalko and Schnitzler 1993). It therefore appears that *P. pygmaeus* is able to persist within urban settings if key habitat features known to be of importance outside of the urban matrix are prevalent. However, caution should be taken in drawing the conclusion that maintaining urban woodland will support *P. pygmaeus* populations given that female *P. pygmaeus* show greater selectivity of foraging locations within this habitat (Lintott et al. 2014).

### The importance of conserving common species

The conservation needs of common species are frequently overlooked given their abundance and widespread distribution (Gaston 2010). However, common species are vital as they contribute strongly to the structure, biomass, and energy turnover of the majority of terrestrial and marine ecosystems (Gaston 2010). Here, we show that bat species previously regarded as relatively common and adaptable to anthropogenic disturbances are still negatively affected by urbanization. Populations of *P. pygmaeus* and *P. pipistrellus* appear to have stabilized (Barlow et al. 2015) after historical declines (e.g., Stebbings 1988), probably as a consequence of increased legal protection, raised awareness of bat conservation, and changes in climate (Barlow et al. 2015). However, our results indicate that increasing urbanization is likely to have a negative effect on both pipistrelle species and therefore support Inger et al. (2015) in their call for an increasing proportion of conservation funds to be spent in ensuring the survival of our common species through the implementation of landscape‐scale environmental improvement programs, such as the creation of effective urban green space schemes. Focusing conservation effort on our commoner species such as *P. pygmaeus* and *P. pipistrellus* will ensure that they avoid a similar fate to the rocky mountain grasshopper (*Melanoplus spretus*) and the passenger pigeon (*Ectopistes migratorius*); common species that were rapidly driven to extinction through anthropogenic activities (Gaston 2010). Additionally, ensuring common species remain with urban landscapes represents one of the best opportunities for the public to encounter and engage with wildlife (Shwartz et al. 2014). Encounters with wildlife may strongly influence attitudes toward conservation, although the majority of studies which validate this hypothesis are primarily descriptive (Shwartz et al. 2014). However, Bjurlin & Cypher (2005) did show a positive relationship between citizen exposure to and appreciation of urbanized kit foxes (*Vuples macrotis*) in California indicating the potential to garner support for wider conservation action and protection of species. Bats are commonly negatively perceived by the public (e.g., Fenton 2003); given the relative frequency of bats found throughout the urban matrix, the opportunity therefore exists to use these encounters as a beneficial mechanism for bat conservation (Bexell and Feng 2013). In this study, we show that whilst both pipistrelle species are relatively widespread within the urban matrix, landscape‐scale environmental programs are still required to ensure that the negative effects of the built environment are minimized.

## Conflict of Interest

None declared.

## Supporting information


**Appendix S1.** The variation in the composition of the landscape of the 124 urban sites that were surveyed.

**Appendix S2.** R2 values obtained from GLM models comparing the percentage of landscape covered by each biotope at a variety of spatial scales to a) the proportion of point counts per transect where *P. pygmaeus* was recorded versus where *P. pipistrellus* was recorded; the percentage of point counts per transect where either b) *P. pipistrellus* or c) *P. pygmaeus* were recorded.
