# Supplementary figures and images for: Differential responses of cryptic bat species to the urban landscape
Source: Ecol Evol. 2016 Feb 26;6(7):2044–52. doi: 10.1002/ece3.1996 (PMC4768637; doi:10.1002/ece3.1996)

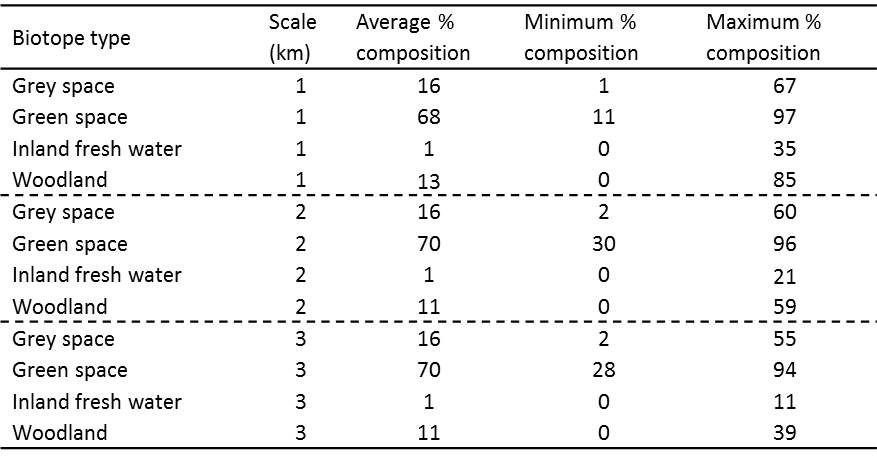

Supplement: Supplementary file 1 — Appendix S1. The variation in the composition of the landscape of the 124 urban sites that were surveyed. [file ECE3-6-2044-s001.jpg]

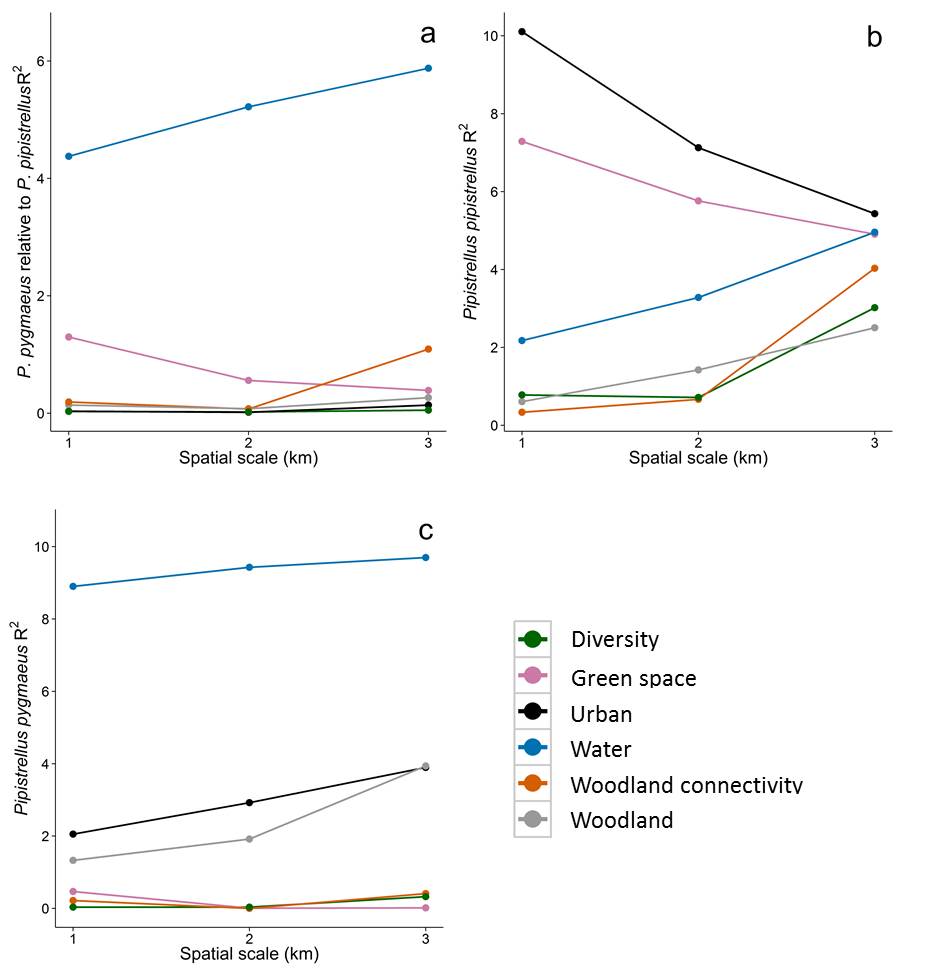

Supplement: Supplementary file 2 — Appendix S2. R2 values obtained from GLM models comparing the percentage of landscape covered by each biotope at a variety of spatial scales to a) the proportion of point counts per transect where P. pygmaeus was recorded versus where P. pipistrellus was recorded; the percentage of point counts per transect where either b) P. pipistrellus or c) P. pygmaeus were recorded. [file ECE3-6-2044-s002.jpg]
